# Will Oral Food Challenges Still Be Part of Allergy Care in 10 Years’ Time?

**DOI:** 10.1016/j.jaip.2023.02.010

**Published:** 2023-04

**Authors:** Nandinee Patel, Wayne G. Shreffler, Adnan Custovic, Alexandra F. Santos

**Affiliations:** a National Heart and Lung Institute, Imperial College London, London, United Kingdom; b Food Allergy Center and Center for Immunology and Inflammatory Disease, Massachusetts General Hospital/Harvard Medical School, Boston, Mass; c Department of Women and Children’s Health (Pediatric Allergy), School of Life Course Sciences, Faculty of Life Sciences and Medicine, King’s College London, London, United Kingdom; d Peter Gorer Department of Immunobiology, School of Immunology and Microbial Sciences, King’s College London, London, United Kingdom; e Children’s Allergy Service, Evelina London, Guy’s and St Thomas’ Hospital, London, United Kingdom

**Keywords:** Food allergy, Oral food challenge, IgE, Basophil activation test, Anaphylaxis, Double-blind placebo-controlled food challenge

## Abstract

Oral food challenges (OFCs) are currently the definitive diagnostic procedure in food allergy. Their design has evolved over the decades to maximize safety, optimize convenience, and address several specific clinical questions. However, they are a resource-intensive investigation that carry a risk for severe allergic reaction in which fatal outcomes, although rare, have been reported. In this review, we explore the many roles that OFC fulfil in the clinical and research settings. We also discuss progress that has been made in developing alternative diagnostic tools and how far these have reached in offering a viable replacement to OFC in clinical practice. Finally, we discuss the ongoing importance of research OFC to improve the future diagnostic capabilities of novel diagnostic tools.

**INFORMATION FOR CATEGORY 1 CME CREDIT**

Credit can now be obtained, free for a limited time, by reading the review articles in this issue. Please note the following instructions.

**Method of Physician Participation in Learning Process:** The core material for these activities can be read in this issue of the Journal or online at the *JACI: In Practice* Web site: www.jaci-inpractice.org/. The accompanying tests may only be submitted online at www.jaci-inpractice.org/. Fax or other copies will not be accepted.

**Date of Original Release:** April 1, 2023. Credit may be obtained for these courses until March 31, 2024.

**Copyright Statement:** Copyright © 2023-2025. All rights reserved.

**Overall Purpose/Goal:** To provide excellent reviews on key aspects of allergic disease to those who research, treat, or manage allergic disease.

**Target Audience:** Physicians and researchers within the field of allergic disease.

**Accreditation/Provider Statements and Credit Designation:** The American Academy of Allergy, Asthma & Immunology (AAAAI) is accredited by the Accreditation Council for Continuing Medical Education (ACCME) to provide continuing medical education for physicians. The AAAAI designates this journal-based CME activity for 1.00 *AMA PRA Category 1 Credit*™. Physicians should claim only the credit commensurate with the extent of their participation in the activity.

**List of Design Committee Members:** Nandinee Patel, MD, PhD, Wayne G. Shreffler, MD, PhD, Adnan Custovic, MD, PhD, and Alexandra F. Santos, MD, PhD (authors); Scott H. Sicherer, MD (editor)

**Learning objectives:**

7. List the different indications for oral food challenges.
8. Explain the benefits and limitations of oral food challenges.
9. Describe possible strategies to reduce the need for oral food challenges.

**Recognition of CommercialSupport:** This CME has not received external commercial support.

**Disclosure of Relevant Financial Relationships with Commercial Interests:** W. G. Shreffler reports royalty payments from UpToDate; grants to his institution from the National Institute of Allergy and Infectious Diseases and Food Allergy Research and Education; sponsored research funds from Aimmune, Angany Therapeutics, Moderna, Regeneron, and Vedanta Biosciences; and consultant fees from Aimmune, ALK, Allergy Therapeutics, Novartis, Regeneron, and Sanofi. A. Custovic reports personal fees Stallergenes Greer, AstraZeneca, GSK, and Worg Pharmaceuticals, outside the submitted work. A. F. Santos reports grants from the Medical Research Council (MR/M008517/1, MC/PC/18052, and MR/T032081/1), Food Allergy Research and Education (FARE), the Immune Tolerance Network/National Institute of Allergy and Infectious Diseases, Asthma UK (AUK-BC-2015-01), the Biotechnology and Biological Sciences Research Council, the Rosetrees Trust, and the National Institute for Health and Care Research through the Biomedical Research Centre award to Guy’s and St Thomas’ NHS Foundation Trust; personal fees from Thermo Scientific, Nutricia, Infomed, Novartis, Allergy Therapeutics, and Buhlmann; as well as research support from Buhlmann and Thermo Fisher Scientific through a collaboration agreement with King’s College London. The rest of the authors and reviewers reported no relevant financial relationships.

## Introduction

Oral food challenges (OFCs), also known as oral provocation tests, are an essential procedure in food allergy in both the clinical and the research settings, serving diagnostic as well as therapeutic purposes in the context of psychotherapy and other aspects of management of food allergy. As the name implies, an OFC is an *in vivo* test during which the patient ingests the suspected food allergen under clinical supervision. Any symptoms and the time relation of symptoms to allergen ingestion are assessed to determine whether and at what dose the patient has experienced a clinically observable allergic reaction or has tolerated the food. The design and location of OFC vary depending on the indication, pre-OFC reaction likelihood, and patient preference.^1^

Double-blind placebo-controlled food challenges (DBPCFCs), in which the assessment is made over two visits with the patient exposed to a placebo on one visit and the suspected allergen on the other, and both patient and clinician are blinded as to the allocation, are still regarded as the reference standard diagnostic tool in food allergy.^2^ They have evolved significantly since the 1950s, when the concept was first suggested.^3^, ^4^, ^5^ However, open food challenges (in which neither patient nor the clinical team are blinded to the doses administered, namely whether active or placebo doses) are now typically used in clinical settings. As the experience of OFC has increased, along with an appreciation of their manifold role in food allergy care, modified challenge protocols have been developed, including single-dose food challenges and interspersed food challenges.^1^^,^^6^ Although OFCs achieve the greatest diagnostic clarity, they are not 100% specific or sensitive. Often, OFCs may not reflect real-world accidental exposures and may carry a risk for allergic reactions including anaphylaxis, and rarely even death.^7^, ^8^, ^9^ Therefore, as other diagnostic tests have advanced,^10^^,^^11^ there has also been a growing desire to minimize the need for OFCs in clinics.

In this article, we review the current utility of OFCs and their relevance for future food allergy care.

## Indictations for OFCS: Why do we need Challenges?

There are several indications for OFCs (Table I), which influence the choice of type of challenge undertaken (Table II).

**Table I:** Samples of indications for hospital oral food challenges

Indication	Reason or example	Settings where used
Investigation or diagnostic procedure		
Diagnostic clarity when history and other testing are equivocal	Lack of recent reaction or uncertain reaction history reduces pretest probabilities and oral provocation becomes necessary	Clinical and research settings
Threshold determination	To assess exposure level required to produce clinically observable allergic reaction (with fixed symptom criteria) at single time point, often before other therapeutic interventions (eg, immunotherapy), after which threshold may be reassessed	Typically used in research trials, increasingly used in clinical settings
Assessment for resolution when history or other testing is suggestive	Downward trends in consecutive sensitization results, in absence of recent high-certainty reactions or history suggestive of non-reactivity upon accidental exposure	Clinical setting
Therapeutic procedure		
Supervised and supported experience of reaction in controlled setting	To confirm ongoing allergy when patient doubts diagnosis and exhibits risk-taking behaviors	Clinical setting
Demonstrating tolerance to low-exposure amounts when allergy is confirmed	When significant anxiety might exist regarding non-ingestion contact or non-contact reactions, or to aid low-level allergen introduction	Clinical setting

**Table II:** Advantages and disadvantages of different aspects of oral food challenges (OFCs)

Aspects of oral food challenges	Advantages	Disadvantages
Oral exposure to food in any setting	Transitions patient from reported knowledge of tolerance to a food to personal experience of tolerance, which is much more likely to result in future incorporation of food into the diet.	Carries risk for life-threatening anaphylaxis. (In the community setting, this can be minimized through careful patient selection and mitigated through patient preparation with comprehensive education on recognition and management of allergic reactions)
In health care setting	Supervision by experienced staff ensures that undue significance is not given to non-specific symptoms whereas highly indicative symptoms are dealt with promptly and not dismissed. Swift escalation of medical treatment is possible when required.	High health care resource use. Anxiety about attending hospital procedures affecting patient behaviors during food challenge.
Blinded to study protocol	Increases objectivity of assessment when pre-probability risk or patient anxiety may bias assessor’s assessment or patient’s experience of placebo symptoms.	Increases resource requirements, halving capacity. Requires significant preparation to mask OFC doses adequately.
Cumulative	Mimics real-world exposure patterns. Increases capacity in allergy centers for low-risk food reintroductions.	Requires careful patient selection to avoid severe reactions.
Incremental	Increases safety of OFC through dose-limitation between observation periods. Allows adaptation of exposure schedule according to reaction history (eg, dosing intervals may be adjusted).	Increases duration of assessment reducing capacity for high volume of visits.

**Figure 1:**
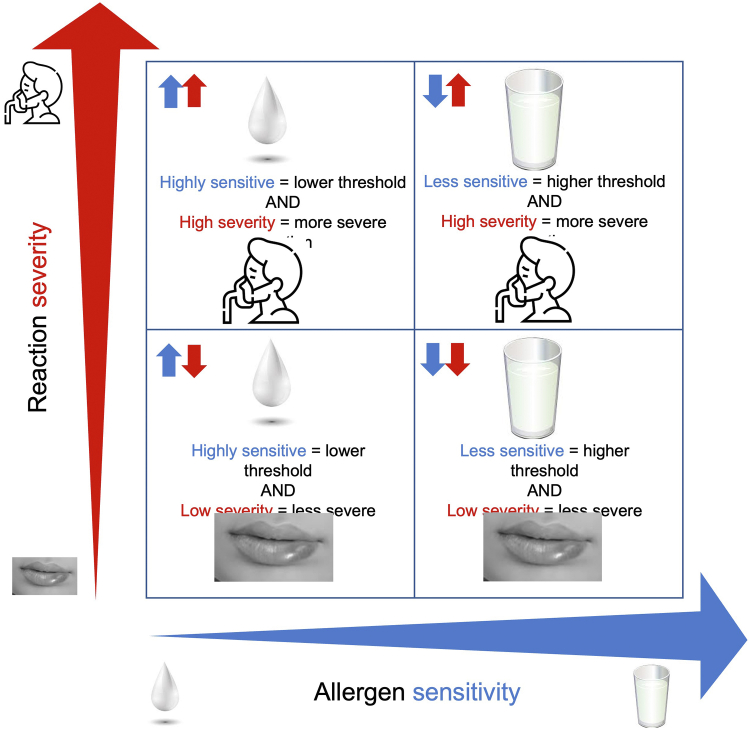
Patient phenotypes: the concept of allergen sensitivity and reaction severity.

In clinical settings, OFCs are used primarily as a diagnostic tool, to confirm or exclude food allergy when a clinical history and assessment of sensitization cannot provide clarity. In addition, OFCs are used to assess for resolution, delabel a food allergy diagnosis, and/or reintroduce the food into the diet. However, OFCs can also provide additional phenotypic information about the allergy, such as sensitivity to the allergen (ie, whether the patient requires a relatively small (higher-sensitivity) or large (lower-sensitivity) allergen exposure to trigger a reaction) or the severity of a reaction (ie, whether the patient experiences few or mild symptoms (low severity) or more, serious, and potentially life-threatening symptoms requiring more treatment (high severity), which can be useful in guiding further clinical management advice (Figure 1).^12^ Finally, OFCs can be used for their experience-based, educational value to support the psychological aspects of managing food allergies, to reduce food allergy–related anxiety and empower patients.

## Oral Food Challenges in IGE and Non-IGE–MEDIATED Food Allergy: Similarities and Differences

The design of OFCs differs when assessing for possible non-IgE–mediated food allergies. Unlike IgE-mediated food allergies, for food protein-induced enterocolitis syndrome there is no broad consensus regarding the framework for dosing. Often, the design of the OFC reflects the timing of reaction ascertained through the clinical history.^13^ Given the length of observation periods in some food protein-induced enterocolitis syndrome challenges, a shift toward multiple-step food challenges in which part of the assessment occurs in the home setting has been proposed, although the safety of this approach has yet to be fully assessed.^14^^,^^15^

In other suspected non-IgE–mediated food allergies such as to cow’s milk, the diagnosis is generally based on elimination of the allergen and its reintroduction over longer periods (typically at least 2-6 weeks). The food of interest is usually administered at home, with support of dietitians when needed, and the patient or family is responsible for observing any time relation between food exposure and symptoms.

Because of the longer interval between exposure and symptoms, the practice is to allow sequential food challenges to occur at home (using food ladders). However, there has recently been an expansion toward using the ladder approach to sequential food challenges in the home setting for some IgE-mediated food allergies in low-risk patients, although it is acknowledged that a robust safety framework (including for patient selection) is needed for this approach, and that it is arguably more akin to slow oral desensitization in its mechanism and often in its clinical intent than it is to an OFC.^16^

## Oral Food Challenges in Research Versus Routine Clinical Practice

In the clinical setting, where OFCs are commonly used to delabel food allergies, open food challenges (typically one to five doses) are the most common and efficient OFC design and can maximize capacity. However, when there has been an equivocal outcome with an open food challenge, DBPCFC can also be helpful.

In research, OFCs have multiple purposes and DBPCFC are more commonly used. Whether for diagnostic or therapeutic clinical trials, DBPCFC provide confirmation of persisting allergy, a definable and sufficiently reproducible reaction threshold, and grade of severity.^17^^,^^18^ They provide higher accuracy in confirming allergy (and therefore eligibility for trial participation) because it is possible to exclude the typically 3% of placebo reactors found with DBPCFC.^19^ However, DBPCFC also have limitations. Blinding to the study protocol becomes redundant in patients known to have a high likelihood of being allergic, who undertake a placebo challenge before the active allergen challenge. In research trials, OFCs generally consist of more doses (up to eight or nine doses in some protocols) and are often repeated at multiple time points to assess the efficacy of an intervention (ie, the change in the threshold after intervention in the active vs placebo intervention groups). Therefore, the consistency of the OFC design throughout the trial is important to compare outcomes and assess the impact of the intervention. However, participants also go through a learning process through the experience of repeated OFCs, which may affect the threshold assessment.^20^

In therapeutic trials for food allergy, the key measure of efficacy has been to assess the reaction threshold and reaction severity through in-hospital OFCs. Although there is a shift toward developing more patient-reported^21^ and real-world outcomes (eg, frequency of community-based accidental reactions),^22^ hospital-based OFCs are likely to remain an important outcome measure in such trials because they allow a controlled, standardized, and reproducible outcome with established feasibility and safety in the research setting.

## Benefits of ORAL FOOD CHALLENGES

Although for some allergens such as peanut other diagnostic tests or a combination of tests can predict the likelihood of a true allergy with a high degree of certainty,^23^^,^^24^ this is not yet possible for the vast majority of food allergens, for which alternative tests leave a high degree of uncertainty, especially in the absence of a clear reaction history.^25^ Therefore, for most allergens, OFCs remain the only definitive way to confirm or refute the diagnosis. Furthermore, even for allergens for which there is evidence about the usefulness of other tests, diagnostic accuracy is limited to populations in which studies have been carried out.^26^^,^^27^

Although the design of OFCs follows a well-established framework focusing on safety and diagnostic clarity,^1^^,^^2^ practical approaches have evolved to improve convenience for patients and families while maintaining safety, and to design individualized protocols according to the purpose of the OFC and pre-OFC reaction risk, while considering the capacity of allergy centers.^28^^,^^29^ In addition, OFCs can be adapted to clarify a diagnosis in specific settings (eg, where only a high-dose exposure has triggered a reaction), where reactions occur only in the context of other cofactors such as exercise, or where delayed responses are suspected,^30^ nuances that cannot be detected by other diagnostic tests. Additional threshold information provided by incremental food challenges may facilitate more active food allergy management strategies in the clinical setting.^31^ This allows guidance on managing precautionary allergen labels and potential low-level allergen introduction based on individual patient response. However, despite careful patient selection, life-threatening allergic reactions remain unpredictable.^32^ Therefore, when oral provocation is required, hospital-based OFCs enable robust procedures for treating severe reactions to be delivered rapidly.^7^

In research, in addition to diagnostic clarity, OFCs provide data on reaction phenotypes (including symptom patterns, organ system involvement patterns, intervals between exposure and symptoms, symptom severity, and individual reaction thresholds or allergen sensitivity). Although there is often the mistaken assumption that the magnitude of results of alternative tests (eg, skin prick test wheal size) are proportional to sensitivity or reaction severity, this is not evidence-based, and it is currently impossible to obtain information about sensitivity and severity without the OFC. In addition, as demonstrated in Figure 1, sensitivity and severity are not directly proportionally related; rather, reaction severity can vary across all degrees of allergen sensitivity. Greater characterization of differences in reaction phenotypes across allergens as well as patient ages, exposure types, and matrices in which the allergen is delivered is essential to improve our understanding of food allergies and inform discussions with patients and other stakeholders regarding individual and scenario-based risks. This information is currently most accurately ascertained through controlled, supervised food exposures^33^ rather than *ad hoc* real-world accidental reaction registries. Only by making such rich datasets available will we be able to develop, validate, and improve alternative diagnostic and prognostic tests. The recent use of large, combined datasets demonstrated promise with some ability to group patients into high or low allergen sensitivity using epitope sensitization patterns^34^ or reaction severity.^35^ Day-to-day variability in the reaction threshold and severity^20^ and the impact of cofactors^36^ are also important to our understanding of how a food allergy diagnosis might translate to the clinical phenotype for any given individual and relate to the real-world future risk. Only by collecting such data will it be possible to assess the potential of alternative tests to predict the key elements of these phenotypes.

Food allergy generates significant psychological burden that is partly related to the fear of severe allergic reactions, although the reality is that life-threatening reactions are extremely uncommon.^37^, ^38^, ^39^ This fear can significantly affect behaviors and interactions regarding food. Thus, separate from their value as an investigation tool in food allergy, OFCs have an important therapeutic role in managing food allergy. For example, in patients with a known food allergy and significant anxiety about a potential reaction to low-dose exposure, contact, or non-contact reactions resulting in significant avoidance behaviors that excessively restricts daily living, an OFC can provide reassurance of non-reactivity or manageable symptoms to low-dose exposures.^12^^,^^40^ Numerous publications demonstrate that this experience of an expected, controlled allergen exposure in a safe setting can reduce food allergy–related anxiety and improve food allergy–related, health-related quality of life.^29^^,^^41^^,^^42^ Improvements can be seen in both the patient and parent (in pediatric food challenges).^43^^,^^44^ Supervised and supported self-administration of adrenaline autoinjector devices after the reaction during OFC can reinforce confidence in the efficacy of self-management of reactions (which is suboptimal in young-people^45^) and can improve health-related quality of life.^46^

In addition, a supervised, supported allergen exposure and reaction can be an educational experience for a patient who is unconvinced about the food allergy diagnosis and has risk-taking behavior.^46^ This can be especially important for the young adult age group, in which many individuals may have no recollection of a previous allergic reactions, leading them to have a degree of skepticism about the diagnosis. They also belong to a group in which risk perception may be changing despite a peak in the risk for fatality from accidental food-allergic reactions at this age range.^47^^,^^48^ However, because OFCs carry the risk for severe allergic reaction, the choice to offer challenges for these indications requires a careful balance of risk and benefit and should always be undertaken jointly with the patient or family.

## Limitations of ORAL FOOD CHALLENGES

Table III lists the main disadvantages of OFCs. This procedure requires extensive resources and expertise, which often are available only to specialized services in hospitals and include a highly skilled clinical team that is clinically trained in this procedure and the treatment of allergic reactions of any severity, including life-threatening anaphylaxis.^2^ Consequently, not all centers or practices are able to offer OFCs. Even in the largest specialized centers, access to OFCs may be limited owing to high demand, which may lead to long waiting lists and lost opportunities in terms of appropriate timing for food introduction. This is especially the case for sensitized infants at high risk for food allergy who are attempting to consume the food for the first time, and for young children who need an OFC to assess possible spontaneous resolution of a food allergy. The main reason for supporting the recommendation to avoid testing infants before introducing allergenic foods in the diet is to avoid a delay in food introduction, because allergen avoidance can increase the risk for developing a food allergy.^49^

**Table III:** Samples of limitations of oral food challenges (OFCs)

Resources to treat allergic reactions of any severity and access to intensive care unit
Highly skilled and trained clinical team
Ideal conditions of OFC impair translation to real-life allergic reactions in community
Potential on-the-day desensitization with sequential dosing during OFC
Anxiety related to fear of experiencing allergic reactions
Need for standardized but food-specific dosing schedule
No absolute accuracy with OFC although it is the reference standard test

Outcomes at hospital-based OFCs cannot completely equate with real-world exposures for some important reasons. First, at OFCs, the dose exposure is incremental, which limits the quantity of allergen exposure needed to trigger symptoms to some degree. However, accidental exposures often lack dose limitations, resulting in larger exposures with the potential for more severe reactions. Second, OFCs are undertaken when patients are well and have good symptom control of other allergic comorbidities. This contrasts with allergic reactions in the community, in which patients may have co-existing co-factors such as intercurrent infection, sleep deprivation, exercise, or active allergic comorbidities (eg, symptomatic asthma) at the time of unplanned allergen exposure, which may facilitate reactions or lead to more severe reactions. In some instances, this may result in a lack of reactivity at the OFC but a reaction to some subsequent home exposure.^50^, ^51^ In addition, the theoretical concept of on-the-day desensitization with incremental allergen exposure at OFCs has the potential to overestimate the reaction threshold or underestimate the reaction severity compared with a single-dose allergen exposure in the community.^52^, ^53^

Oral food challenges often create significant anxiety among patients, their families, and even health care professionals.^54^ Patients may worry before or during the OFC (as sequential doses are eaten, or as symptoms commence and the clinical team surrounds the patient to monitor and assess allergic symptoms), or after the OFC, if the patient experiences an allergic reaction. Anxiety is an important component of the negative impact of food allergy on patients and their families. Thus, particularly for some families, an alternative that does not require the additional anxiety of OFCs may be desirable.^43^^,^^44^

Oral food challenge guidelines often offer indicative dosing schedules that are general and applicable to all foods.^2^ However, one size does not fit all when it comes to food protein per portion. Although it is important to standardize dosing schedules, individual doses need to be specific for each food and to be adjusted to portion sizes and age. For instance, certain foods are low in protein content, and some teenagers or adults may eat much more of the food than the average portion.

Although they are the reference standard, OFCs retain an error rate. If one considers post-OFC follow-up, about 3% of results are false-positives, approximately 3% are false-negatives, and about 9% are inconclusive (the patient experiences only subjective symptoms, or perhaps the child refuses to eat the food, resulting in an incomplete OFC).^55^, ^56^, ^57^, ^58^ Finally, OFCs are time-consuming and affect school or work attendance for the patient and carer. They require experienced staff, which can affect the availability of time and staff for other resource-intensive procedures.

## Alternatives to ORAL FOOD CHALLENGES

In routine clinical practice, OFCs are reserved for equivocal cases due to the risk of allergic reactions and the limited resources compared with demand. Consequently, the diagnosis and/or monitoring of food allergy resolution is made based on the history and evidence of allergen-specific IgE as much as possible (Table IV). As many sensitized individuals are tolerant to foods, in the absence of a history of an allergic reaction to the food in question, the use of validated cut-offs with high sensitivity and a high positive predictive value can be helpful to diagnose a food allergy.^10^^,^^59^ Extrapolation from published studies to clinical practice is most accurate when study populations mirror the full breadth of the clinic population. Good examples are the 95% positive predictive value (PPV) for peanut allergy using a skin prick test (8 mm) or a specific IgE (15 kU/L) result that was validated in the United Kingdom and United States in different studies performed years apart, and therefore can be used reliably in these populations.^60^, ^61^, ^62^

**Table IV:** Possible strategies to reduce number of oral food challenges without reducing diagnostic accuracy

Strategy	Guidance
Skin prick test	>95% positive predictive value cutoff = likely allergic especially if history of allergic reaction; allergen avoidance should be recommended 95% negative predictive value – 95% positive predictive value cutoffs = likely allergic if history of reaction; equivocal if no history, in which case challenge should be recommended <95% negative predictive value = likely tolerance, especially in absence of history of allergic reaction, in which case food consumption can be attempted
Specific IgE to extracts	
Specific IgE to allergen components	
Peptide-specific IgE	
Basophil activation test	
Home introduction of food	In absence of history of reaction and of evidence of allergen-specific IgE

An IgE to individual allergen components for certain foods can be more specific than a specific IgE to whole allergen extracts and reduce the need for OFCs (eg, peanut Ara h 2, hazelnut Cor a 14, cashew Ana o 3) or encourage the performance of OFCs and reduce food allergy overdiagnosis.^61^ Similarly, the use of the basophil activation test (BAT) can reduce the number of reactive OFCs and encourage the performance of OFCs in sensitized individuals with favorable serology (eg, levels below the 95% PPV cutoff) and a negative BAT.^63^ Basophil activation tests examine the functional characteristics of IgE.^64^ In peanut allergy, the optimal cutoff for BAT that was determined in a study published in 2014 had high PPV (95% to 100%) and high negative predictive value (90% to 98%), and these were confirmed in external validation studies.^35^^,^^61^ This suggests that BAT may accurately predict whether a child would react to the allergen during OFCs. However, no test is absolute; like any test, such a result needs to be interpreted in the clinical context of the individual patient being tested.

Although alternative tests facilitate the assessment of the presence or absence of a food allergy in larger volumes of patients than would be feasible with OFCs, they are unable to provide information regarding the sensitivity to an allergen (eg, reaction thresholds) and have only limited capacity to inform conversations about disease severity (eg, a peanut-allergic patient with Ara h 8 predominance may be more likely to have a pollen food-allergy syndrome diagnosis with limited local allergic symptoms compared with a patient with Ara h 2 sensitization). Data are emerging about the potential ability of sensitization and functional *in vitro* testing to provide information regarding allergen sensitivity and reaction severity.^34^^,^^35^ However, these findings need additional validation, and it is unlikely that in isolation, allergy tests will be able to address all of the information needs of patients in the absence of experiential knowledge (eg, accidental or supervised exposure), something demonstrated to enhance management efficacy and confidence.^29^^,^^65^ There is the potential, however, for these enhanced tests to be used in combination with tailored supervised food exposures to optimize the patient’s diagnostic journey and resource use. *In vitro* tests can be used initially to inform patients about the likelihood of allergy, the estimated threshold level, or the phenotype, and then risk-stratified tailored supervised food exposure can reinforce and enhance this understanding to empower patients through self-experience. In selected patients, this approach may allow more active food-inclusion advice.^31^ Moreover, although neither OFC nor alternative tests allow prognostic information about disease trajectory, longitudinal patient assessment using both modalities has the potential to improve the prognostic ability of alternative tests in the future.

**Figure 2:**
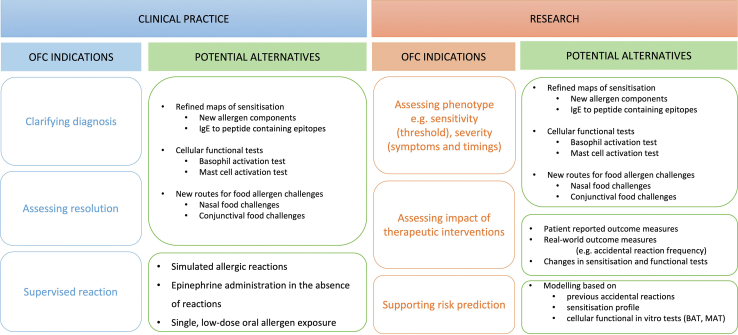
Changing utility of oral food challenges (OFCs) and possible alternatives. *AAI*, adrenaline auto-injector; *BAT*, basophil activation test; *MAT*, mast cell activation test.

Alternative *in vivo* provocation tests to hospital-based OFCs include home introductions and hospital-based supervised feeds (ie, cumulative OFCs). Home introductions are often undertaken after allergy tests in case of the low likelihood of allergy (eg, in patients with no history of allergic reaction to the food in question and no evidence of IgE-sensitization to that food, provided atopic allergic comorbidities are well-controlled). They include specific recommendations regarding the amounts that should be reached within a certain time (eg, ladders, introduction of food using age-appropriate portion on 1 day). Supervised feeds are essentially a cumulative OFC (single dose corresponding to an age-appropriate portion or a set target dose, as opposed to an incremental dose protocol OFC) undertaken in patients with either a high likelihood of tolerance (eg, more commonly used in infants in the context of early introductions to prevent allergy) or a reassuring risk profile (eg, previous high-threshold reaction with evidence of potential disease resolution). Both alternatives increase the clinic capacity for managing a higher volume of patients, but they require careful patient selection to minimize patient risk. Figure 2 summarizes potential alternatives to OFCs in clinical practice and research.

## Patient Preferences Regarding Oral Food Challenges

Unfortunately, there are few systematic reviews in the published literature about patient preferences regarding OFCs, and much of what is reported is from the provider’s perspective rather than directly from patients. As discussed earlier, there is ample evidence of anxiety as a significant comorbidity of food allergies and OFC as a procedure is both the focus of anxiety and a potential means of its reduction. Those are not mutually exclusive groups: that is, the same patient with a high level of anxiety about OFC may achieve a significant reduction in anxiety from OFCs, though it is our clinical impression that patients vary in motivation to pursue OFCs and that motivation from the same patient or caregiver may change with age or other circumstances that is not limited to changes in history or testing with a bearing on the actual likelihood for tolerating the food (eg, school attendance, new siblings, child autonomy, motivation to consider treatment options). That variation in motivation is reflected in a small survey study by Kraft and colleagues,^66^ in which they identified aggressive versus conservative patterns of patient and caregiver motivation - those that were more aggressive were more likely to undergo OFC for peanut or tree nut and tended to be less likely to have experienced a reaction historically.^29^^,^^37^ We and others have observed a growing trend toward patient interest in low-dose challenges. Rationale for these limited-dose challenges may include increase the confidence regarding food allergy management (eg, less anxiety about precautionary labeled products by virtue of tolerating a low but substantial amount without symptoms)^29^ or support of introducing low-dose food with immunotherapeutic intent,^67^ an intervention that appears to be growing in clinical practice even as it is being systematically evaluated (eg, NCT03907397).^67^ Because of the wide range of patient preferences, goals, and potential trade-offs associated with OFC, it follows that as long as OFCs are indicated, the shared decision-making framework is ideal whenever OFCs are discussed.^68^ Finally, given the opportunity, patients consistently tell us that an alternative to OFCs is an important unmet need.

## Conclusions

Double-blind placebo-controlled food challenge is the reference standard procedure for food allergy diagnosis. Open challenges have been adopted as the practical equivalent in clinical settings. Clinical services seeing patients with suspected food allergy should be able to offer OFCs in a feasible and timely fashion. As diagnostic tests improve, they can be used to predict clinical reactivity and reduce the need for OFCs (especially reactive OFCs) and increase the accuracy and response to demand, reduce anxiety, and improve the experience of patients and their families. In clinical practice, one may be able to avoid OFCs in patients by confirming food allergy through combining the clinical history with allergy test results or using high PPV test results (eg, specific IgE to components, BAT) when using appropriate cutoffs for the local population. However, any tests need to be interpreted considering the clinical context of individual patients.

In answer to the question of whether OFC will still be part of allergy care in 10 years’ time: yes, given their clinical value, we are likely to still be doing OFCs in 10 years. However, our aim should be to reduce the need for diagnostic OFC markedly by developing more robust and accurate tests that do not carry the risk associated with anaphylaxis. The same goal should exist for using OFCs to monitor the response to immunomodulatory treatments. However, substantial further OFC data (with an array of OFC designs) will likely be needed over the next decade to characterize aspects of reaction phenotypes and dose distributions better for two purposes: to provide insights into the characteristics of food allergic reactions by allergen and provide large controlled datasets through which novel allergy tests can be validated. As these data emerge and the use of these new tests for disease diagnosis and prognosis prediction become more robust, the requirement for clinical OFCs for diagnostic purposes should be drastically reduced with OFCs remaining available for selected purposes.
